# The Status of Human and Animal Fascioliasis in Iran: A Narrative Review Article

**Published:** 2015

**Authors:** Keyhan ASHRAFI

**Affiliations:** *Department of Microbiology, School of Medicine, Guilan University of Medical Sciences, Rasht, Guilan Province, Iran*

**Keywords:** Human fascioliasis, Animal fascioliasis, Epidemiology, Lymnaeid snails, Iran

## Abstract

**Background:** The public health importance of human fascioliasis has increased during last few decades due to the appearance of new emerging and re-emerging foci in many countries. Iran, as the most important focus of human disease in Asia, has been included among six countries known to have a serious problem with fascioliasis by WHO. Various aspects of the disease in Iran are discussed in this review.

**Methods: **This narrative review covers all information about human and animal fascioliasis in Iran, which has been published in local and international journals from 1960 to 2014 using various databases including PubMed, SID, Google Scholar, Scopus, Science Direct.

**Results: **During the period of the study the infection rates of 0.1% to 91.4% was noted in various livestock. Despite the higher infection rates of livestock in southern areas in past decades, human disease has been mostly encountered in northern Provinces especially in Guilan. Recent studies indicate noticeable decrease in prevalence rates of veterinary fascioliasis in Iran, however the prevalence rates of fascioliasis in livestock in northern Provinces of Guilan and Mazandaran seem to remain at a higher level in comparison to other parts. New foci of the disease have also been reported recently.

**Conclusion: **While the prevalence of animal fascioliasis has decreased during last decades, human fascioliasis emerged as a public health problem in the country. The validity of new foci of human fascioliasis needs complementary standard studies.

## Introduction

Fascioliasis the disease caused by two liver flukes of the Genus *Fasciola, F. hepatica *and *F. gigantica,* was considered mainly as an important veterinary problem until the end of 1980s, because of the substantial production and economic losses it causes in livestock (1). During this period, human fascioliasis has been considered a rare disease, concerning just sporadic cases reported from different geographical zones (2). 

The public health importance of human fascioliasis increased following the record of 2594 infected individuals in 42 countries of all continents from 1970-1990 (3). In recent years the number of human cases affected by *Fasciola *spp. has increased drastically with estimated ranging from 2.4 million (4, 5) to 17 million people infected (6). These figures might be even underestimated due to lack of knowledge about the situation of the disease in humans in many African and Asian countries (7).

Appearance of many areas having been described as endemic for human fascioliasis with prevalences and intensities ranging from low to very high, has resulted in a new picture of the disease, from an almost exclusive veterinary problem to both veterinary and medical importance (8-10). In Asia, human fascioliasis is mainly encountered in the Islamic Republic of Iran and at a lower level in Vietnam. WHO has recently included Iran among six countries, which are known to have a serious problem with fascioliasis (11). Implementation of control strategies in each endemic zone needs basic information about different aspects of the disease especially the epidemiological characteristics and transmission routes. 

The purpose of this review was to collect all available information including those, which have been published in scientific local journals in Persian, which is not usually usable for foreign researchers and other people who are interested in the issue, especially local health responsible, parasitologists and WHO expert members. Here we discuss the current and past status of the disease in various parts of Iran.

## Methods

This narrative review covers all information about human and animal fascioliasis in Iran that has been published in local and international journals up to 2014. The relevant articles were obtained by searching human fascioliasis, animal fascioliasis, epidemiology, molecular characterization, lymnaid snails, experimental infections, fascioliasis treatment, slaughtered livestock and Iran as keywords. The publications having any information about the scope of the present study were included. Various databases including PubMed, SID, Google Scholar, Scopus, and Science Direct were used for collecting the present data (1960-2014).

***Guilan Province, northern Iran***

Until 1989, human fascioliasis was sporadic in Iran and several cases of human infections have been reported from different parts of the country. Following the first report of human fascioliasis from thyroid of an Iranian patient, several sporadic cases of hepato-biliary and cutaneous fascioliasis demonstrated in separated geographical zones (12, 13). 

An unexpected event happened in 1989 in Guilan Province, which changed the picture of the disease not only in Iran but also in whole Asia. Thousands of individuals with signs and symptoms manifest mainly as epigastric and right upper quadrant pain, fever, chill, sweating, weight loss, urticarial and chest signs accompanied with high eosinophilia attended in medical centers of the Province during a short period. In fact, it was the onset of the first largest ever outbreak of human fascioliasis in the world, involving up to 10000 individuals in Bandar-Anzali and Rasht districts of Guilan Province (14, 15). 

The second outbreak that happened some 10 years later left about 5000 infected people in the same region. Hundreds of human cases have also been recorded between two outbreaks and thereafter in local health centers. Bandar-Anzali appears to be the most important endemic zone including most of the human cases during the epidemics and inter-epidemic periods.

Due to its unique epidemiological characteristics a specific pattern of transmission has been proposed for Caspian Sea areas named "Caspian Pattern" define as a hypoendemic area with large-scale epidemics sometimes affecting more than 10000 people (16). The numbers of fascioliasis cases in Guilan Province recorded following the second outbreak are summarized in Table 1.

**Table 1:** Number of human cases based on information present in local health centers of Guilan Province, before and after the second outbreak

**Year**	**Number of human cases in different localities of Province**				
	**Bandar-Anzali**	**Rasht**	**Lahijan**	**Other districts**	**Guilan Province**
1998	258	330	6	10	604
1999	2465	1691	17	21	4194
2000	1161	114	14	17	1306
2001	348	81	59	13	501
2002	445	91	16	24	576
2003	143	32	1	3	179
2004	331	146	19	14	510
2005	190	31	16	15	252
2006	63	0	6	2	71
2007	33	40	6	11	90
2008	50	12	7	1	70
2009	46	1	35	4	86
2010	22	11	15	6	54
2011	18	9	13	4	44
2012	16	16	24	13	69
2013	11	11	7	2	31
2014	21	9	19	3	52
Total	5621	2625	280	163	8689

***Mazandaran Province, northern Iran***

Mazandaran province has located in southern littoral of Caspian Sea, adjacent to the eastern areas of Guilan Province. The spur of Alborz Mountains towards the sea at the level of Mazka area has divides the Province into two zones, west and east Mazka. The western areas of Mazandaran show more similarity to Guilan Province in terms of rainfall and people traditions, and this issue has affected the distribution of human and animal fascioliasis in the Province. The prevalence of fascioliasis in western Mazandaran is clearly higher than those of eastern parts. 

A total of 107 human cases have been diagnosed during 1997-2002 period in Mazandaran, most of which been reported in western areas (17). The gender and age distribution of human fascioliasis in Mazandaran is similar to those in Guilan Province. The infection has been detected in almost all age groups and there is no significant difference between two genders. 

The prevalence of animal fascioliasis in some areas of western Mazandaran reached 75% while it was only 27.3% in eastern parts. Fascioliasis is more prevalent in cattle than sheep, but sheep present higher intensities of infection. Buffaloes are seen in some areas of Mazandaran but when present, they show high infection rates. The infection rate of fascioliasis in goats is lower than sheep and cattle and it seems they play a secondary role in the disease transmission (17). 

***Kermanshah Province, western Iran***

The first documented case of human fascioliasis in Kermanshah Province belongs to a 23-year old woman from Sahneh district, adjacent to Kangavar, with manifestations of a subcutaneous painful mass (18). In 1998, a small outbreak of human fascioliasis happened in Kangavar district located in western Province of Kermanshah. The clinical and serological survey demonstrated seventeen positive cases among the population of Cheshmeh- Derazeh village. This outbreak verified a new emerging focus of human fascioliasis in western part of the country (19). A 10- year follow up (1998-2008) in Kermanshah Province has also demonstrated 17 other cases from different localities of the Province, especially in Kermanshah district, the capital of Kermanshah Province. 

***Kohgyluyeh va Boyerahmad Province, southwestern Iran***

A new focus of human fascioliasis has also been introduced recently from Yasuj distric, Kohgyluyeh va Boyerahmad Province (20). Personal observations of some local physicians concerning the suspicious human cases in the area, accompanied with high prevalence rate of *F. hepatica* in local livestock encouraged local parasitologists to perform a study for determining the seroprevalence rate of the infection. Anti-*Fasciola* antibodies were detected in sera of 1.8% of individuals by ELISA technique. All sero-positive individuals had the history of consuming wild freshwater plants (20). For verification of this area as a focus of human fascioliasis, stool examination of randomly selected local population is needed. On the other hand, serological tests alone are not sufficient for verification of active infections and introducing new foci of the human disease.

***Ardabil Province, northwestern Iran***

In 2010 and 2011, two cases of human fascioliasis were reported from Ardabil Province, northwestern Iran (21, 22). The first case, a 6-year old boy, who was seropositive for visceral leishmaniasis and did not properly answer to anti Kala-azar therapy, diagnosed as a case of fascioliasis by finding *Fasciola* eggs in liver sections. The second was a 79-year old man with gastrointestinal disturbance and symptoms of intestinal obstruction. Several peritoneal mass were noted at surgery, in which large numbers of *Fasciola* egg observed. 

Following these findings, a seroprevalence study performed on 458 sera for detecting the rate of human fascioliasis in the Province. Using ELISA technique, the rate of 1.96% was reported (23). The stool samples of rural and urban communities should be examined by appropriate methods for determining the real situation of the disease in this area. 

***Recent case reports***

In addition to abovementioned foci, having higher numbers of human cases, the awareness of Iranian physicians and parasitologists has resulted in report of several cases of the disease from various localities all over the country. These cases include cutaneous (18, 24), peritoneal (22), hepato-biliary (25-28), eye (29), hepatic abscess (30, 31), ascites (32, 33) and multi organ involvement (34). 

***Gender and age effects in human fascioliasis in Iran***

The gender and age effect in fascioliasis has been investigated in some endemic regions worldwide with various results. In hyperendemic areas females shed more eggs than males but prevalences do not differ between both sexes, while in mesoendemic regions of Egypt the prevalence in females appeared to be statistically significantly higher than in males (35-37). 

Study of human population in endemic region of Guilan in different situations indicated various results. Studies which have been conducted during the first outbreak of human fascioliasis in Bandar-Anzali revealed the prevalences of 50% and 36.5% using serological and coprological examinations respectively (15, 38). Recently, a community-based study has been performed in this region to identify the real prevalence of human fascioliasis in a non-epidemic situation (39). The prevalences of 0.4% and 1.2% were indicated using coprological and serological methods (CL1 ELISA) respectively. This result demonstrated a hypoendemic situation in endemic zone of Bandar-Anzali. 

The studies performed during first human outbreak in Guilan verify a statistically significant difference between two genders (15, 38) while in non-epidemic situations the difference between two sexes was not significant (*P*>0.05) (39). The similar picture was also indicated in Mazandaran, Kohgyluyeh va Boyerahmad and Kermanshah Provinces (17, 19, 20). 

It appears that the gender role in fascioliasis in Iran and Egypt seems to be very similar because of close cultural and social behaviors. In rural communities of both endemic zones, females involve in agricultural tasks, meal and salad preparation and washing activities. Since, the numbers of infected cases in endemic regions of Iran are very lower than that of Egypt (hypoendemic versus mesoendemic respectively) the difference between gender and age have not yield a statistically significant result. 

The age effect in fascioliasis in Iran seems to be interesting. During the outbreaks happened in Guilan and Kermanshah the highest number of infected individuals were seen in lower age groups; 10-19 years in Kermanshah (19) and 10-29 years in Guilan (15, 38). In comparison, in non-epidemic situations, the numbers of infected cases were higher in age groups above 20 years old (17, 39). This issue might be correlated with adult activities, because they are more involved in agricultural and home tasks while their children are at school and have lower contact with contaminated materials and environments. On the other hand, during epidemic situations all age groups are at risk of infection through eating large scale contaminated freshwater plants. 

**Sources of human infections**

Human infections usually take place through ingestion of infective metacercariae attached to the leaves of aquatic plants. Although several contamination sources for human infections have been proposed, ingestion of metacercariae infected wild freshwater plants (generally called watercress) is the main route of human contamination. Many species of freshwater plants are responsible for human infections in different geographical zones, some of which are not necessarily parts of the usual human diet (7, 10, 17). 

Bargues et al. showed that 13% of the metacercariae of all isolates in Bolivian Altiplano are floating and demonstrated the role of metacercariae contaminated natural water in human contamination (40). Recently, Ashrafi and Mas-coma highlighted various sources of the infection, which expose travelers and migrants at risk of human fascioliasis in different parts of the world and proposed fascioliasis to be included in the list of diseases of importance in Travel Medicine (41).

***Guilan Province***

Several species of wild grown aquatic and/or semi-aquatic plants (*Eryngium* spp. and *Mentha* spp.) are main part of usual human diet in many areas of Iran, especially in endemic region of Guilan Province, the most important zone of human fascioliasis in the country. Three species of *Mentha pulegium*, *Mentha piperita* and *Eryngium caucasicum* are the main species that have been implicated in transmission of human fascioliasis in Guilan endemic zone by previous workers (14, 15, 38). 

The villagers collect these aromatic plants and present them beside the streets and in traditional markets throughout the year. These vegetables are very popular and may be eaten fresh or ground and mixed with walnuts, various spices, garlic and fresh olives for the preparation of an appetizer called "Zeitoon-Parvardeh" or may be used along with a great quantity of salt for the preparation of a herbal paste called "Delar". The amount of salt, which is used for Delar preparation is very high, which is the basis of the local name “Green Salt”.

The impact of ingredients using for preparation of these local foods on viability and infectivity of liver fluke metacercariae have been evaluated. The results indicated the possibility of human contamination following consumption of these traditional foods, when prepared with fresh vegetables presenting attached metacercariae (42, 43). 

***Other Provinces***

In other parts of the country, from which cases of human fascioliasis have been reported, several species of aquatic plants are implicated in transmission of the disease. *Nasturtium microphyllum* (locally named "Boolaghuti") and *Mentha longifolia* in Yasuj district, in southwest of Iran (20), *Eryngium* spp. and *Mentha* spp. in Mazandaran Province at littoral of Caspian Sea (17),* Nasturtium* spp. and *Falcaria vulgaris* (localy named "Paghaze") in Kermanshah Province, western Iran (18) are the main plants implicated in transmission of human infections. 

***Snail intermediate hosts***

Snails of the family Lymnaeidae are of great parasitology importance, because of their capacity to act as intermediate hosts for numerous trematode parasites including those of medical and veterinary impact (44). Several species of the Genus *Lymanea* are main intermediate hosts for transmission of human and animal fascioliasis in endemic regions, worldwide. It is well known that the two-fasciolid species show different lymnaeid snail host specificity:* F. hepatica *is mainly transmitted by species of the *Galba/Fossaria* group, whereas *F. gigantica* is transmitted by species belonging to the *Radix* group. 

Several species of *Lymnaea* including *L. (Radix) auricularia*, *L. (Stagnicola) palustris*, *L. schiraziensis*,* L. pregra*,* L. stagnalis, L. gedrosiana* and *Galba truncatula*, have been reported from different Provinces of Iran (17, 45-53). It appears that *L. gedrosiana* as the main intermediate host of *F. gigantica* and *L. truncatula,* the principal intermediate host of *F. hepatica* present throughout the country.* L. stagnalis*, which has been shown to have the potential for transmitting both fasciolids, found mainly in Khuzestan, Lorestan, Charmahal va Bakhtiari and western Azerbaijan Provinces. Experimental infections and field observations have demonstrated the capacity of Iranian lymnaeids in transmission of human and animal fascioliasis.

***Fasciola*** **spp. and Lymnaeid snails in experimental infections**

The susceptibility of different Iranian species of *Lymnaea,* to *F. hepatica* and *F. gigantic*a investigated by some researchers (54-57).* Galba truncatula* is the main species belonging to the intermediate snail host species of *F. hepatica* where present (58-60) and experimentally it appears to also be very susceptible for Iranian *F. hepatica* isolates (54). 

Arfaa et al. demonstrated the role of *L. gedrosiana* in transmission of *F. hepatica* in Iran (55). Cruz-Reys and Malek have also verified the susceptibility of Iranian *L. gedrosiana* to *F. hepatica* infection with an infection rate of 32.5% (61). Massoud and Sadjadi demonstrated the susceptibility of *L. gedrosiana* and *L. pregra* to infection with *F. gigantica* and resistance of them to *F. hepatica* infection and did not accept the results of Arfaa et al. because they believed that those researchers have dealt with *F. gigantica* ova (54, 55). 

Rohani and Massoud studied the susceptibility of *L. gedrosiana* to various numbers of *F. gigantica* miracidia and showed the infection rate of 69% when using the exposure dose of 10 miracidia per snail (56). Ashrafi et al. also verified *L. gedrosiana* as a potent intermediate host for transmission of *F. gigantica* in the endemic zone of Guilan Province by comprehensive experimental studies (62). 

Although there are some disagreements concerning the role of this snail in transmission of *F. hepatica* in Iran, all abovementioned workers verify this snail as a potent intermediate host for *F. gigantica*. Shahlapour studied the susceptibility of *L. palustris* and *L. stagnalis* to miracidia of *F. hepatica* and *F. gigantica*. Results indicated that very young *L. palustris *snails are susceptible to *F. gigantica*, while *L. stagnalis* was susceptible to both *F. hepatica* and *F. gigantica* miracidia (57). Metacecaria obtained from experimental infections of those snails caused fascioliasis in rabbits and sheep.

Moreover, recent studies suggest that* F. gigantica* may be the predominant fasciolid species in Guilan, a fact supported by morphology of liver flukes found in livers of slaughtered livestock and the widespread distribution of *L. gedrosiana* throughout the endemic areas (45).

***F. hepatica*** **and** ***G. truncatula*** **in natural infection**

Ashrafi et al. also reported *G. truncatula* naturally infected with *F. hepatica* larval stages from mountainous areas of Talesh district, northwest of Guilan Province (46). Nuclear ribosomal DNA ITS-2 sequences proved that they were *F. hepatica* and *G. truncatula*. The liver fluke ITS-2 sequence was identical to that of *F. hepatica* from Spain and the Northern Bolivian Altiplano and that of *G. truncatula* to the haplotype H-2 known in Portugal, Spain, France and The Netherlands.

The author has also demonstrated the natural infections of *L. gedrosiana* by *F. gigantica* in endemic zone of Bandar-Anzali (63). All these information verifies the potential role of *G. truncatula* and *L. gedrosiana* in transmission of human and animal fascioliasis in endemic zone of Guilan.

***Trends of distribution of lymnaeid snails in Guilan Province***

Most cases of human fascioliasis in Iran have been reported from endemic region of Guilan, mainly in Bandar-Anzali and Rasht districts, including thousands of cases in two largest ever outbreaks in the world. This endemic zone comprises of flatlands, adjacent to the Caspian Sea, and highlands far from the Sea. 

The highest numbers of human infections in Guilan have been detected in people who live in lowlands of Bandar-Anzali and Rasht districts, in some places even below the sea level. Throughout these areas *L. gedrosiana*, *L. palustris* and *L. schiraziensis* are prevalent while *G. truncatula* rarely encountered. Close to the mountainous areas, at foothills,* G. truncatula* appears to present in small bodies of superficial clean and cold waters originated from the adjacent mountains, and in mountainous regions *G. truncatula* seems to be the only prevalent intermediate snail. This picture is the same in other parts of the Province (63).

The distribution of lymnaeid snails in different localities of Guilan are in accordance with that of *F. hepatica* and *F. gigantica* in local livestock. In lowlands, surrounding Bandar-Anzali and Rasht, the high incidence rate of fascioliasis by *F. gigantica *in local cattle verifies this fact. Of 928 adult liver flukes collected from 13 infected livers of cattle, in Rasht and Bandar-Anzali slaughterhouses, 91.1% were diagnosed as *F. gigantic*a and 8.9% as *F. hepatica*.

The situation in mountainous areas is quite different. More than 90% and 99% of trematodes recovered from sheep and goats respectively, were diagnosed as *F. hepatica.* On the other hand, cattle is the most prevalent livestock in flatlands of Guilan and mainly infected with *F. gigantica* while in highlands, in which *G. truncatula* is prevalent, the highest population of livestock belong to sheep mainly infected with *F. hepatica* (45, 63). 

Another snail, which is widespread in flatlands of Guilan, is *L. schiraziensis*. This snail is genetically distant but phenotypically very close to *G. truncatula*. Recent studies have demonstrated that *L. schiraziensis* is not implicated in transmission of human fascioliasis in its widespread geographical zones (53).

***Molecular identification of Lymnaid snails in endemic regions of Iran***

In recent years, some molecular tools have been developed in relation to nuclear ribosomal and mitochondrial DNA sequences, which provided valuable information for clarifying the classification of snails within family lymnaeidae (44, 53, 64). 

Sequences of the second transcribed spacer of the nuclear ribosomal DNA (rDNA ITS-2) indicated that the *L. gedrosiana* from Guilan Province of Iran is identical to the sequence of *Radix auricularia *populations of the genotype GT-1 known in the Czech Republic, Austria and United Kingdom available in the GenBank (Accession Number AJ3119628), that of *L. truncaula* from Guilan identical to the sequence of *G. truncatula* of the genotype GT-2 known in Europe (Accession Number AJ296271) and finally, the sequences of *L. palustris* of Guilan is identical to that of *Lymnaea (Stagnicola) palustris* found in population of Normandie (france), Bavaria (Germany) and Friesland (The Netherlands) available in GenBank (Accession Number AJ319620) (63). 

Salahi-Moghaddam et al. proved the presence of three species of lymnaeids in northern Province of Mazandaran by genetic analyses (65). These snails including *Lymnaea (Stagnicola) Palustris*, a secondary intermediate host of *F. hepatica*; *G. truncatula*, the main intermediate host of *F. hepatica*; and *Radix gedrosiana*, the main intermediate host of *F. gigantica*. Based on the results of this study, *G. truncatula* presents different rDNA ITS-1 and ITS-2 genotypes, which could explain the variability detected in Mazandaran.

***Geographical distribution of fasciolids in Iran***

Two *Fasciola* species, *F. hepatica *and *F. gigantica*, are involved in both animal and human fascioliasis (9). Fascioliasis due to *F. hepatica *is a great health problem in many countries with temperate climates such as in Europe, the Americas and Australia, whereas the major endemic areas for *F. gigantica *are large tropical regions of Africa, and many areas of Asia including Uzbekistan, Turkmenia, Iran, Iraq, India and Pakistan, etc. (7, 16). 

In Asia and Africa, the distribution of *F. gigantica *and* F. hepatica *overlaps and this overlap makes it difficult to identify the particular species involved in human infections, so that it is often referred to as *Fasciola* sp. (58). In general, in tropical countries when both species coexist, *F. gigantica *is usually endemic in lower regions while *F. hepatica *is endemic in the highlands (66, 67). 

A similar picture occurs in Iran, where the distribution of *F. gigantica* and* F. hepatica *overlaps in almost all areas and both species may simply be obtained from a single definitive host. Concerning humans, the fluke species involved in human fascioliasis remains to be identified, having been referred to as *F. hepatica* by some researchers*.*

A phenotypic study of fasciolid adult flukes from naturally infected bovines from Guilan was carried out by means of an exhaustive morphometric analysis using traditional microscopic measurements and an allometric model. The Iranian fasciolids were compared to *F. hepatica* and *F. gigantica* standard populations, i.e. from geographical areas where both species do not co-exist (Bolivia and Burkina Faso, respectively). Results obtained revealed that Iranian *F. hepatica-like* specimens are larger than the *F. hepatica* standard and Iranian *F. gigantica-like* specimens are longer and narrower than the *F. gigantica* standard, but with smaller body area. In the Iranian fasciolid populations analyzed, the morphometric and allometric study shows that intermediate forms are also present. The allometries of fasciolid intermediate forms described for the first time in this study (68). 

Very recently, Ashrafi et al. have determined the distribution of *F. hepatica* and *F. gigantica* in endemic area of Guilan and explained the relationship between zonal overlap and phenotypic traits. The results of the present study demonstrated that, in Guilan, fascioliasis follow a zonal overlap transmission pattern with *F. hepatica*-like transmission taking place mainly in the highlands and *F. gigantica*-like transmission mainly in the lowlands (69). 

In past decades, high prevalence and intensities of fascioliasis have been reported among domestic animals in Khuzestan Province, southwestern Iran (70). The results of these studies also verified the higher infection rate of *F. gigantica *in comparison to* F. hepatica*. In another study conducted in six Provinces of Iran both species were observed in the livers of slaughtered animals in five Provinces located in distinct geographical zones, while all parasites obtained from infected livers of slaughtered animals in Khuzestan Province was *F. gigantica* (71).

The distribution of *F. hepatica* and *F. gigantica* also overlap in other parts of Iran in which cases that are more human have been reported, including Mazandaran, Kermanshah and Kohgyluye va Boyerahmad Provinces (17, 20, 52). 

***Molecular identification of fasciolids***

Ashrafi studied the molecular characteristic of fasiolids in Guilan Province of Iran using the sequences of second transcribed spacer of the nuclear ribosomal DNA (63). Based on the results the rDNA ITS-2 of *F. hepatica* from Guilan is identical to the sequence of *F. hepatica* from Europe available in the GenBank (Accession Number AJ272053) and the sequences of *F. gigantica* from Guilan to that of *F. gigantica* from Burkina Fasso available in the GenBank (accession Number AJ853848).

In another study, samples from buffaloes and goats from different localities of northern Iran were genetically characterized by sequences of the first and second Internal Transcribed Spacers (ITS-1& ITS-2) of nuclear ribosomal DNA (rDNA). Comparison of the sequences of Iranian samples with sequences of *Fasciola* spp. from GenBank indicated that the examined specimens had sequences identical to those of the most frequent haplotypes of *F. hepatica* (n=22, 45.83%) and *F. gigantica* (n=17, 35.42%), but differed from each other in different variable nucleotide positions of ITS region sequences, and their intermediate forms (n=9, 18.75%), which had nucleotides overlapped between the two *Fasciola* species in all the positions (72). Molecular identification of fasciolids in Northwestern Province of Zanjan has also demonstrated the presence of *F. hepatica* in the area (73).

Mahami-Oskouei et al. studied 90 *Fasciola* isolates from three Provinces of Khorasan, Fars, and Eastern Azerbayjan using PCR-RLFP method (71). Seventy isolates were diagnosed as *F. hepatica* originated from three Provinces and twenty isolates as *F. gigantic*a from Khorasan and Fars Provinces. In another study, both species of *F. hepatica* and *F. gigantica* were genetically identified in Eastern Azerbaijan using ITS-1 marker (74). Recently, in addition to *F. hepatica* and *F. gigantica* genotypes, the first molecular evidence of an intermediate genotype of *Fasciola* has also been demonstrated in Fars Province (75).

Rokni et al. analyzed 50 *F. hepatica* and 30 *F. gigantica* isolates from sheep, goats, cattle and buffaloes of three Provinces of Tehran, Khuzestan and Western Azerbaijan by a simple PCR-restriction method (76). All samples from Khuzestan Province, southwestern Iran, showed ITS-1 sequences similar to *F. gigantica* from China, while all samples from Western Azerbaijan, northwestern Iran, and Tehran Province, north of the central plateau of Iran, had ITS-1sequences similar to *F. hepatica *of many other parts of the world. 

**Animal Fascioliasis**

The oldest reports of animal fascioliasis in Iran belong to Sabokbar and Sabbaghian who demonstrated high prevalences of infection in livestock of Guilan and Khuzestan Provinces respectively (77, 78). In recent years, several studies have been performed in different localities of the country to clarify the prevalences and intensities of animal fascioliasis. Here, the mean prevalence rates of animal fascioliasis in different localities of Iran are presented in brief, and the comprehensive information is available in Table 2.

***Northern Provinces***

The studies which performed from 2004-2007 indicate the prevalence rates of 9.35-32% for sheep and 32.1-55% for cattle in Guilan Province (45, 79-81). 

**Table 2:** Prevalence rates of fascioliasis in livestock from different provinces of Iran

**Area**	**Prevalence rate in livestock (%)**				**References**
	**Sheep**	**Cattle**	**Goat**	**Buffalo**	
**Northern Provinces**					
Guilan	32	32.1	-	17	79
	-	32.1	-	-	80
	9.53	32.5	-	-	81
Mazandaran	7.3	25.4	-	50	17
	7.8	12.1		-	81
	1.2	3	-	-	71
Golestan	2.5	3.1	-	-	81
Tehran	31.2	25.5	64.3		88
	2.01	2.2	-	-	83
	2.07	-	-	-	82
Semnan	0.1	-	-	-	82
**Northwestern Provinces**					
Eastern Azerbaijan	1.62	-	-	-	86
	8.57	-	-	-	86
	1.7	1.1	-	-	71
Western Azerbaijan	-	-	-	38.7	89
	12.12	-	-	-	87
Ardabil	20	-	-	-	83
	5.3	25.9	4.9	11.4	84
	21.6	25.35	-	-	85
Zanjan	1.96	-	-	-	82
Qazvin	1.14	-	-	-	82
**Western Provinces**					
Ilam		8.5	-	-	90
	0.57	3.14	0.49	-	91
Lorestan	2.6	2.8	2.6	-	93
	9.5	-	-	-	84
Kordestan	2.91	-	-	-	82
Kermanshah	13.4	9.5	6.9	-	52
Hamadan	4.9	9.5	4.5	-	94
	4.13	-	-	-	82
Markazi	1.73	-	-	-	82
	0.56	0.57	-	-	95
	0.9	1.1	-	-	71
**Southwestern Provinces**					
Khuzestan	35	54	-	57	78
	29	49.2	11.2	91.4	70
	-	-	-	13.09	97
	1.2	4.5	-	-	71
	0.9	5.3	2.3	10.4	98
	0.4	5.5	-	-	99
KohgyluyevaBuyerahmad	11.75	12.5	7.16	-	96
CharmahalvaBakhtiari	4.1	-	-	-	84
**Southestern Provinces**					
Kerman	2.1	-	-	-	83
	3.7	6.9	3	-	100
Fars	1.07	0.59	0.24	-	101
	0.6	0.4	-	-	71
Hormozgan	2.49	-	-	-	82
Bushehr	0.65	-	-	-	82
**Central Provinces**					
Esfahan	4.03	-	-	-	82
Yazd	1.37	-	-	-	2
	1.47	-	-	-	94
**Eastern Provinces**					
Northern Khorasan	0.42	0.73	0.2	-	98
	0.35	0.71	0.2	-	102
Razavi Khorasan	0.7	0.7	-	-	71
**Whole country**					
Iran	2.49	-	-	-	82
	19	17.8	11.5	18.2	90

These rates were 1.2-7.8% for sheep and 3-25.4% for cattle of Mazandaran Province (17, 71, 81). In another Northern Province, Golestan, the infection rates of 2.5 % and 3.1% have been reported for sheep and cattle respectively (81). In Tehran and Semnan Provinces the prevalence rates for sheep was 0.1 and 2.07% respectively (82, 83).

***Western Provinces***

Reports from northwestern Provinces indicate the prevalences of 1.14-21.6% for sheep, 1.1-25.9% for cattle, 11.4-38.7% for buffaloes and 4.9% for goats (71, 82, 84-89). The infection rates of fascioliasis in livestock of Western Provinces were 0.56 -13.4% for sheep, 0.57-18.7% for cattle and 0.49- 6.9% for goats (52, 90-95). Prevalences of 0.4-35% for sheep, 4.5-54% for cattle, 10.4-91.4% for buffaloes and 2.3-11.2% for goats have been reported from southwestern parts during past decades (70, 71, 96-99).

***Central and Southern Provinces***

Results of several studies in central and southern parts of the country revealed the prevalence rates of 0.6-3.7% for sheep, 0.4-6.9% for cattle and 0.24-3% for goats (71, 82, 100, 101).

***Eastern Provinces***

**Fig 1:**
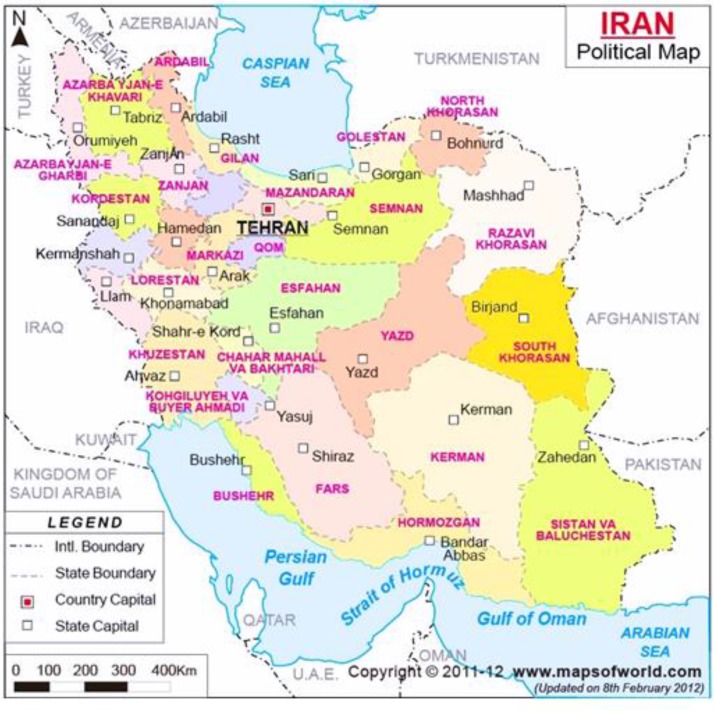
Geographical situation of different provinces on Iran political map

In eastern and northeastern Provinces the prevalence rates of 0.35-0.7% for sheep, 0.7-0.73% for cattle and 0.2% for goats have been reported (71, 98, 102). The geographical situation of all Provinces is shown in political map of Iran, Fig. 1.

***Clinical manifestations in Iranian patients***

Fascioliasis usually manifests in two clinically phases; acute and chronic. In general, the most important consequences of the disease are hepatic lesions, chronic inflammation of bile ducts and fibrosis. The major symptoms of acute phase are fever, abdominal pain, gastrointestinal disturbances, urticaria, and respiratory symptoms while in chronic phase biliary colic, epigastric pain, fatty food intolerance, nausea, jaundice, pruritus and right upper-quadrant abdominal tenderness are the most important clinical manifestations of the disease (1, 96, 97, 103, 104).

***Patients from Guilan Province***

During the first outbreak of human fascioliasis in Iran the clinical manifestations of the disease was investigated by study of 100 infected individuals. In the first stages the main signs and symptoms were as followings: fever (77%), weight loss (88%), chill (83%), sweating (83%), epigastric pain (87%), right upper quadrant pain (79%), joint pain (77%), right shoulder pain (67%), neck pain (69%), anorexia (75%), cough (75%), dyspnea (57%), chest pain (61%), urticaria (32%), hepatomegaly (20%), splenomegaly (5%) and cutaneous lesion (5%). In later stages of the disease most of the abovementioned signs and symptoms subsides while epigastric and right upper quadrant pain continued (38).

***Kermanshah and Yasuj Provinces***

In the small outbreak occurred in Kermanshah Province the main clinical manifestations of fascioliasis were weight loss (47%), epigastric pain (41%), abdominal pain (29%), right upper quadrant pain (24%), fever, chill, headache, anorexia, chest pain (18%), low back pain, coughing and itching (12%), myalgia, neck pain, dyspnea and urticaria (6%), hepato-splenomegaly (29%), right hypeochondria tenderness and sweating (24%), tachycardia (18%) (19). In Yasuj, abdominal pain, allergic manifestations and headache was seen in some seropositive cases (20).

***Sporadic cases***

In some sporadic cases of the disease reported from various parts of the country the following signs and symptoms have been reported: right upper quadrant and epigastric pain, fever, anorexia, weight loss, urticaria, hepatomegaly, body pain, chill, sweating, jaundice and nausea (24-27, 105). In addition, some rare manifestations such as cutaneous painful mass (24), peritoneal (22), eye (29), spleen, pancreas and kidney involvement (34), and liver abscess (30) have been reported. In most of the cases, eosinophilia higher than 20% was also reported. 

**Diagnosis**

Fascioliasis might be diagnosed in different stages of the disease by various methods. In chronic phase, diagnosis is mainly carried out by detection of parasite ova in stool or biliary aspirates, while serological tests are routinely used for detection of anti-*Fasciola* antibodies in serum samples in acute phase and in ectopic fascioliasis as well. Immunological methods are also suitable for diagnosis of chronic fascioliasis by detecting specific antigens in stool samples and antibodies in the serum. 

ELISAs are the most commonly used serological tests, with sensitivities ranging from 92-100% and specificities ranging from 84-98% (106-108). Serological tests, such as Fas2-ELISA or CL1-ELISA, are able to detect the circulating IgG antibodies elicited by infected individuals against *Fasciola* Excretory-Secretory antigens (Fas2 and CL1) secreted by juvenile and adult worms (109). The antibodies to Fas2 and CL1 rise rapidly by a few weeks of infection, which indicates the high antigenecity of these antigens. 

***Diagnostic measures in Iran***

***Clinical diagnosis***

Since fascioliasis was not a medically important and prevalent disease in Iran until 1989, the outbreak of a human disease with clinical picture of hypereosinophilia in Guilan Province, was first ascribed to Visceral Larva Migrans, probably due to the report of some cases of the disease in the area and cross-reaction of serological tests. Removing adult *Fasciola* from gallbladder of a local patient, suffering from a liver disease, during the surgery attracted the attention of local specialists to the possibility of fascioliasis outbreak. This important finding directed the clinicians to the correct diagnosis, but the absence of a reliable serological test in that situation forced the physicians to rely on a presumptive diagnosis of the disease based on epidemiological, clinical and hematological features.

For this reason, the Iranian Ministry of Health established an instruction for dealing with the patients suspected to be infected with fasioliasis. Based on this instruction, following three conditions might be encountered:

*•* *Suspected cases*. Diagnosis in this situation should be mainly based on the clinical history, including fever, right upper quadrant pain and liver enlargement.

*•* *Probable cases*. History of clinical manifestations accompanied with eosinophilia of 20% or higher and a positive serological test if present.

*•* *Definite case*s. History of clinical manifestations and observing the parasite eggs in stool sample.

Based on this instruction probable and definite cases were considered as positive and treated with triclabendazole. 

***Serological diagnosis***

During the first outbreak of fascioliasis in Guilan serological diagnosis was carried out using ELISA and Counter-Current Immune Electrophoresis (CCIE). Fifty percent of the samples using ELISA techniques and 34.95% using CCIE were positive indicating that point prevalence of the disease was in Anzali district. Excretory- secretory antigens were used in ELISA technique in this study and it was 52.6% more sensitive than CCIE (*P*<0.001). In this study, 52.4% of ELISA positive cases were negative by CCIE technique and only 47.4% of cases were positive by both methods. Based on the results the specificity of ELISA technique was 22.5% less than CCIE (15).

O'Neill et al. developed an ELISA test for diagnosing human fasciolosis in an endemic area of Northern Bolivia. The assay was based on the detection of serum antibodies reactive with antigens secreted by the parasite. The sensitivity and specificity of this ELISA test were analyzed on 176 patients residing in the Guilan Province. The ELISA employed total molecules secreted by the parasites (excretory-secretory, ES, products) and a protease, termed cathepsin L1 (CL1), which was purified from this preparation, as antigen. Using this assay, both CL1 and ES exhibited a sensitivity of 100% (all 176 patients tested positive) and a specificity of 100% and 98.9%, respectively (108). The authors concluded this standardized diagnostic ELISA as a valuable tool to diagnosis human fascioliasis in Iran and could be employed in a large survey to determine the prevalence of the disease throughout this region. 

Rahimi et al. established a Fast-ELISA method and evaluated its efficacy versus Standard-ELISA for diagnosis of human fascioliasis in Iran (110). The sensitivity, specificity; positive, and negative predictive values were detected as 97.2%, 100%, 94.6%, and 95.6% for both tests. Cut-off values, sensitivity, specificity, and other important parameters of the two evaluated tests determined no sensed difference between two methods. 

***Imaging techniques***

Radiographic and imaging techniques have been used for diagnosis of fascioliasis with variable sensitivity (111-113). Sonography appears to be more sensitive than computed tomography in the chronic phase because some specific characters including thickening of the major bile ducts, motile or dead parasites within the ducts or gallbladder, mild dilatation and edema of the biliary ducts are readily detected by sonography (114, 115). 

Alizadeh et al. reported Endoscopic Ultrasonography (EUS) as a noninvasive excellent method for diagnosis of biliary fascioliasis and showed that it is as accurate as magnetic resonance cholangiopancreatography (MRCP) and endoscopic retrograde cholangiopancreatography (ERCP). They proposed EUS as selective method when the need for therapeutic intervention is not likely (116). On the other hand, ERCP should be used only when the clinical probability for choledocholithiasis is high. Mansour-Ghanaei et al. described the sonographic findings of hepatic lesions in 248 patients with fascioliasis from endemic region of Guilan Province (117). The authors verified sonography as a useful method to confirm hepatobiliary lesions in human fascioliasis, particularly in endemic situations. 

***Parasitological techniques***

 Stool examination, by Kato-Katz technique, is one of the main tools for diagnosis of human infection in chronic stage. Kato-Katz technique is a quantitative and simple method suitable for field studies and situations when dealing with large number of fecal samples, but it is not sensitive enough. For this reason, more than one stool examination is needed to increase the sensitivity of the test. Coprological methods are unable to diagnose infection in the incubation and acute phases, so the infection might be missed within 3-4 month after exposure and in ectopic fascioliasis. Kato-Katz method has been frequently used for diagnosis of human fascioliasis in Guilan endemic areas using the kits donated by WHO (14, 38, 39). This technique is very useful for egg count and determining the intensity of the infection. Cup-sedimentation is a sensitive but time-consuming method and is not appropriate when dealing with large number of stool specimens. 

**Treatment**

***Use of bithionol***

Several drugs have been used for treatment of human fascioliasis during recent decades. Bithionol proved to be a potent fasciolicidal drug with minimal side effects and proposed as the drug of choice by some researchers (118-121). In endemic region of fascioliasis in Iran bithionol (40 mg/kg for 15 days) was used for treatment of 31 patients during the first outbreak of human fascioliasis with 66-69 percent effectiveness but 60% of the patients were hospitalized due to severe drug side effects (122). The necessity for multiple dose, long period of treatment and need for medical surveillance during treatment with bithionol, has led to test more effective and safer drugs, including triclabendazole.

***Clinical trial of triclabendazole in Iran***

Triclabendazole, a benzimidazole derivative, has been routinely used since 1983 in veterinary medicine to control infections with *Fasciola* spp. in domestic herbivorous animals. The first clinical trial of Triclabendazole for treatment of human fascioliasis was carried out in endemic regions of Iran based on WHO protocol using veterinary formulation of triclabendazole, Fasinex^®^ (123). This study comprised of a randomized clinical trial (quazi-experimental survey) and a historical cohort, lasted for 6 years. Based on the results of this study the efficacy of triclabendazole was proved to be 94%, along with good tolerance and minimum side effects, it was recommended as the drug of choice for treatment of human fascioliasis. 

In another randomized trial a single, double and triple dose of 10 mg/kg of a human formulation of triclabendazole, Egaten^®^, was evaluated. One hundred sixty five patients aged 10-65 years who were referred to Bandar-Anzali health center for treatment were included in this study. The results showed the safety and efficacy of 10 mg/kg of the drug for 1-3 days in the treatment of patients with fascioliasis. The cure rate was not significantly different (*P*>0.05) among the three dose groups (124). At present, treating of the cases of human diseases in Iran performs by using Egaten^®^ (human pharmaceutical preparation of triclabendazole) which is donated by WHO. This drug is available in health centers of Guilan endemic regions and other endemic parts of the country and prescribed to the patients with confirmed fascioliasis. 

***Use of Metronidazole***

The effectiveness of metronidazole in treatment of human fascioliasis was first evaluated by Nik-Akhtar & Tabibi in Iran (125). Mansour-Ghanaei et al. studied the effect of metronidazole in treating human fascioliasis in Guilan Province (126). Forty-six patients who included in this study was egg positive and had abdominal pain, sometimes accompanied with other symptoms. The patients received 1.5 g/day metronidazole orally for 3 weeks. All patients became pain free after treatment and the stool exam was negative in 76.1% of treated individuals. Based on the results, metronidazole (1.5 g/day for 3 weeks) appears to be an effective, available and well-tolerated drug, which could be regarded as an alternative treatment in fascioliasis. 

***Use of Praziquantel***

Although praziquantel is highly active against almost all trematodes and cestodes, its inefficacy for the treatment of human fascioliasis has been reported in various countries (120, 127-129). Yadegari et al. also administered praziquantel (70 mg/kg) for treatment of one hundred infected cases, with 2% cure rate proving ineffectiveness of this drug for treatment of human fascioliasis (130). 

***Evaluation of Triclabendazole drug resistance***

To evaluate the situation of drug resistance in Guilan Province, Hosseini et al. studied the efficacy of triclabendazole using for treatment of fascioliasis in sheep (131). One hundred forty two egg positive sheep in test group and five sheep in control group received 10 mg/kg triclabendazole and placebo respectively. All infected animals which received triclabendaz-ole were egg negative (epg=0) while the fecal samples of control group were still positive for *Fasciola* ova. This study verified the 100% efficacy of triclabendazole as the choice drug for treatment of fascioliasis in the region.

**Prevention and control**

Recent research has shown that human endemic areas present different transmission and epidemiological patterns (5, 10). Strategies for control should be designed based on the epidemiological characteristics of the disease in each endemic country. In hyperendemic zones of Bolivia and Peru, fascioliasis transmission is so intense that tens of thousands of people are estimated to be infected, particularly children. In this situation they have decided to treat all school-age children living in the endemic areas once a year and free-of-charge (11).

 In mesoendemic zone of Egypt, the strategy of selective treatment is used and triclabendazole is distributed free-of-charge to all school-age children who test positive during mass screenings in the villages (11, 132, 133). In Vietnam, which is a hypoendemic region a simple passive case finding approach, has been selected for control of human fascioliasis. In this situation, only those people who are symptomatic and attend at health centers receive triclabendazole. 

***In Iran***

In endemic region of Bandar-Anzali in Guilan Province, northern Iran, in which the largest ever outbreaks of human fascioliasis have occurred, a community based epidemiological study on human fascioliasis has recently been performed. Based on the results of this study human fascioliasis is hypoendemic in the region (39). 

Occurrence of large periodic outbreaks, occasionally involving up to 10000 people in Guilan Province accompanied with the presence of *F. hepatica* and *F. gigantica* and several *Galba-Fossaria*, *Radix* and *Stagnicoline* lymnaeids in the area, has resulted to define a pattern of fascioliasis transmission for northern part of Iran locating at littoral of Caspian Sea, named "Caspian Pattern". Therefore, based on the cultural and epidemiological characteristics of the region and WHO proposal, the following measures are performed for controlling of human fascioliasis in Guilan Province:

1. A passive case finding approach, which contains treatment either of infected people who test positive or on a presumptive basis, using triclabendazole.
2. Establishment of effective veterinary public health measures, including regular treatment of livestock with Fasinex®.
3. Awareness of people living in endemic areas about the danger of eating raw, uncooked aquatic and semi-aquatic plants. For this purpose the educational programs should be provide by health centers and broadcast by mass media to decrease the risk of human infections, especially in high risk seasons. This item was very useful during the outbreaks of fascioliasis in Guilan Province for decreasing the chance of human infections.
4. Since raw wild grown fresh water plants are a part of human diet in Guilan, especially in endemic areas, culture of popular plants in safe places (home gardens) will definitely decrease the chance of infection. This manner is now uses by some residents living in the endemic areas of Guilan Province.

***Concluding remarks***

Veterinary fascioliasis has been prevalent in Iran, at least during past fifty years, with high prevalences and intensities especially in southern and northern parts, at the littoral of Persian Gulf and the Caspian Sea. Despite the higher infection rates of livestock in southern areas in past decades, human disease has been mostly encountered in Northern Provinces especially in Guilan. Recent studies indicate noticeable decrease in prevalence rates of veterinary fascioliasis in Iran, mainly due to the interaction of veterinary organizations by treatment of livestock and increased awareness of local farmers. 

Nevertheless, based on recent studies, the prevalence rates of fascioliasis in Northern Provinces of Guilan and Mazandaran seem to remain at a higher level in comparison to other parts. On the other hand, while the prevalence of fascioliasis in livestock of southern Iran has decreased, the higher prevalence rates are still reported from Northern Provinces in addition to higher human cases. It appears that various environmental and social factors involve in this issue, some of which are as followings:

1. High amount of rainfall in Guilan Province especially in Bandar-Anzali. The amount of rainfall in Guilan Province is about 1200-1800 mm, which is about 4-5 times higher than the average of the country. Bandar-Anzali has the highest rate of rainfall in Iran, the mean rainfall of this city in last fifty years has been 1850 mm, and in some years, it even passed 2000 mm.
2. Appropriate temperature and humidity. Bandar-Anzali has the most humid climate of any city in Iran, having a climate somewhat similar in its heavy autumn and early winter rainfall and persistent high humidity and low sunshine to the Sea of Japan Coast of Japan. The average of annual temperature and humidity is about 13.2-19.2 C and 76-88% respectively. These conditions are very appropriate for existence of parasites and their intermediate hosts.
3. Food habits. It appears that eating habit of Guilan populations has a critical role in their infection with fascioliasis. Eating raw aromatic wild grown freshwater plants is very popular in Guilan inhabitants. These vegetables are available throughout the year in traditional markets of endemic areas. This picture is almost unique of northern coasts of Caspian Sea, especially in Guilan Province.
4. Presence of numerous water collections and irrigation canals rich in aquatic vegetation. These water resources, which persist throughout the year, provide appropriate conditions for lymnaeid existence and liver flukes transmission.
5. Release of local livestock in harvested rice fields and pastures in rural areas and suburbs. These animals play a crucial rule in distributing of parasite eggs and snail infections.
6. Presence of different species of lymnaeid snails. The snail intermediate hosts present in endemic areas most of the year in parallel with the distribution of both fasciolids and their definitive hosts.

Finally, based on present data, transmission of veterinary fascioliasis is taking place in different area of the country, while appropriate conditions for establishment of human fascioliasis exist only in a few geographical zones, especially in Guilan Province. Although various studies have been performed on fascioliasis in endemic regions of the country, many disease aspects have remained to be clarified. Determining the species involved in human infections, factors involved in two large outbreaks in Guilan, establishment of the dynamics of fascioliasis, determining the season or seasons of transmission are among those issues need to be investigated in future. 
